# Laparoscopic adjustable gastric band in an obese unrelated living donor prior to kidney transplantation: a case report

**DOI:** 10.1186/1752-1947-4-107

**Published:** 2010-04-19

**Authors:** Anoop N Koshy, Stephen Wilkinson, Jeff S Coombes, Robert G Fassett

**Affiliations:** 1 Department of Medicine, University of Tasmania, Launceston General Hospital, Launceston, Tasmania, Australia; 2 Department of Surgery, Royal Hobart Hospital, Hobart, Tasmania, Australia; 3 School of Human Movement Studies, The University of Queensland, St. Lucia, Queensland, Australia; 4 Department of Renal Medicine, Royal Brisbane and Women's Hospital, Brisbane, Queensland, Australia; 5 School of Medicine, The University of Queensland, Brisbane, Queensland, Australia

## Abstract

**Introduction:**

Obese living donors who undergo donor nephrectomy have higher rates of intra-operative and post-operative complications. Many centres exclude obese donors from living donor transplant programs. Diet, exercise and medication are often ineffective weight loss interventions for donors, hence bariatric surgery should be considered.

**Case presentation:**

We report the case of a 53-year-old Caucasian woman who underwent laparoscopically adjustable gastric banding. The procedure enabled her to lose sufficient weight to gain eligibility for kidney donation. After losing weight, she had an uncomplicated laparoscopic donor nephrectomy surgery, and the recipient underwent successful kidney transplantation.

**Conclusion:**

Laparoscopically adjustable gastric banding should be considered for obese potential living kidney donors whenever transplantation units restrict access to donor nephrectomy based on the increased surgical risk for donors.

## Introduction

Obese living donors are often excluded from surgery because of the associated increased risk of local wound complications and blood loss [1,2]. Consequently, obese patients are categorized as high-risk and hence excluded from donor nephrectomy surgery by many centers even though equivalent mortality, non-wound related complications and recipient renal outcomes have been recorded across different body mass index (BMI) groups [3,4].

Laparoscopic adjustable gastric banding (LAGB) surgery involves the placement of a silicone band around the proximal part of the stomach through laparoscopy, which reduces the volume of food that a patient can ingest. Many obese patients are unable to reach a suitable weight via traditional methods such as diet, exercise or medication, and LAGB should thus be considered as an option. We have reported cases of successful weight loss associated with LAGB in patients with end-stage kidney disease (ESKD). To the best of our knowledge, however, this is the first report where this weight loss technique was specifically applied to lose weight to enable kidney donation [5]. Hence, we report the case of an obese donor who underwent LAGB to achieve weight loss that was not possible with diet and exercise alone. The success of the subsequent donor nephrectomy surgery and kidney transplantation suggests that LAGB should be considered more often in patients with a similar condition.

## Case presentation

A 59-year-old Caucasian man with autosomal dominant adult polycystic kidney disease presented with progressive chronic kidney disease (CKD) in late 2006. He started peritoneal dialysis, which was changed to haemodialysis after a severe episode of peritonitis. His blood group was A-negative and his BMI was 24 kg/m^2^.

**Figure 1:**
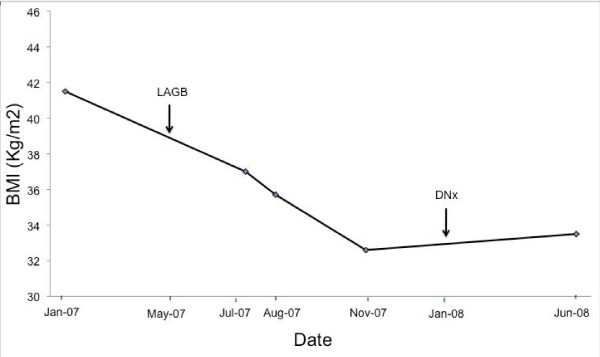
**Changes in our patient's body mass index prior to and following LAGB surgery and donor nephrectomy**.

Our patient's 53-year-old Caucasian wife whose blood group was O-positive was evaluated as a potential living unrelated kidney donor. Results of their T and B cell ALLO cross-matches were both negative. Both patients were CMV-negative and they had a 5-antigen mismatch. She underwent satisfactory nephrological and cardiovascular system evaluation. However, on evaluation by a transplant surgeon, the donor did not meet the eligibility criteria for surgery due to her morbid obesity. She had a BMI of 41.5 kg/m^2^, (weight = 130 kg). She had a history of hysterectomy and smoking. After several failed attempts at losing weight by conventional weight loss methods such as diet and exercise, she underwent LAGB in May 2007. Figure 1 shows that after the LAGB, our patient's BMI decreased from 41.5 kg/m^2 ^to 32.6 kg/m^2 ^over 7 months. This equated to 21.5% loss in her original weight and an excess weight loss (calculated with a BMI of 25 kg/m^2 ^as the reference point) of 54%. She subsequently gained eligibility for donor nephrectomy.

A BioEnterics^® ^LAP-BAND^® ^was used on our patient. The key advantage of this band lies in its inner tubing. The tubing is connected to a reservoir under the skin of the abdomen and can be accessed by inserting a needle, thus allowing for a non-invasive increasing or decreasing of the liquid within the balloon, which in turn either tightens or loosens the LAGB.

An uncomplicated left laparoscopic hand-assisted donor nephrectomy was eventually performed on the donor (wife) in January 2008. The transplant operation on the recipient (husband) was complicated by intra-operative hypotension, which required fluid resuscitation and his admission to the intensive care unit of our hospital. A post-transplant MAG 3 renal scan showed good perfusion of the transplanted kidney, and a post-reperfusion kidney transplant biopsy showed a viable kidney with well-preserved glomerular and tubular morphology. Eight months following the donor nephrectomy, the donor's kidney function was stable and her LAGB was still in place. Moreover, her most recent BMI is 33.5 kg/m^2^.

## Discussion

Over 60% of Australian adults are overweight or obese and the current prevalence of obesity is 2.5 times higher than it was in 1980. A similar trend is likewise being observed in most industrialized countries [6]. Such a marked increase in the prevalence of obesity, coupled with the success of live donor transplantation and a shortage of deceased donor organs, has forced a re-examination of donor acceptability criteria, as well as a move to relax these criteria in order to include donors with a BMI of >30 kg/m^2^.

Obesity has been shown to contribute significantly to the risk associated with developing cardiovascular disease, diabetes, dyslipidemia and hypertension [7]. The main contraindication for morbidly obese donors is an increased incidence of intra-operative and post-operative complications [8]. A study by Mendoza *et al. *reported an overall complication rate of 30% in patients undergoing laparoscopic urological procedures, including nephrectomies where the mean BMI of patients was 35.1 kg/m^2 ^[8]. Other studies have also reported longer operative times, an increased rate of conversion to open surgery, increased wound complications, and surgical blood loss in obese donors [1,3,8]. Even with an increased risk of developing complications in obese patients, most studies conclude that obesity should not be a contraindication for laparoscopic donor nephrectomy primarily due to equivalent rates of morbidity, mortality and recipient renal outcome [3,4]. However, in a retrospective study on kidney transplant recipients, Kandapara *et al. *reported a lower mean glomerular filtration rate at 12 months among those who received a cadaveric kidney from overweight or obese donors than those from normal BMI donors [9].

Glomerular hyperperfusion and hyperfiltration as physiological adaptation from afferent arteriolar vasodilatation in obesity are proposed mechanisms of pre-transplant kidney damage in the donors [9]. This raises concerns regarding the long-term graft function among recipients from obese donors, as well as the long-term renal function of obese individuals who undergo donor nephrectomy.

In our reported case of LAGB prior to donor nephrectomy, our patient experienced a weight loss of 28 kg, which was a 21.5% reduction of her original weight. Subsequently, she was able to gain eligibility to the surgical transplant program and successfully donated a kidney to her husband. To the best of our knowledge, this is the first reported use of LAGB in this situation. Reported complications of this procedure include band slippage, gastric pouch dilation, infection, and a mortality rate of 0.53% [10]. Most of these complications can be managed by band removal or adjustment [11]. Other bariatric surgical procedures such as Roux-en-Y gastric bypass and vertical banded gastroplasty can also be considered. Similarly, Branco *et al. *reported the successful use of Roux-en-Y gastric bypass in two patients prior to successful laparoscopic donor nephrectomy where both donors lost over 30% of their initial weight and had uneventful post-operative courses [12]. However, a systematic literature review revealed that the mortality rate associated with Roux-en-Y gastric bypass is 10 times higher than with LAGB and six times higher than with vertical banded gastroplasty [13].

The advantages of LAGB include the minimal invasiveness of the procedure, reduced post-operative pain, and low rates of associated morbidity and mortality [10]. Other reported benefits of LAGB are a complete remission in Type 2 diabetes mellitus (64%), resolution of gastroesophageal reflux disease (89%), and improvements in the quality of life of patients [14].

## Conclusion

We report the successful use of LAGB in a morbidly obese donor to enable her eligibility for a laproscopic hand-assisted nephrectomy and successful recipient kidney transplantation. LAGB should be considered for obese potential living kidney donors whenever transplantation units restrict access to donor nephrectomy based on their increased surgical risk for donors.

## Abbreviations

BMI: body mass index; CKD: chronic kidney disease; CMV: cytomegalovirus; DNx: donor nephrectomy surgery; ESKD: end-stage kidney disease; LAGB: laparoscopic adjustable gastric banding.

## Competing interests

The authors declare that they have no competing interests.

## Authors' contributions

ANK reviewed our patient's history and wrote the first draft of the manuscript. SW performed the LAGB surgery. JSC reviewed the manuscript and provided editorial assistance. RGF served as our patient's nephrologist. He also reviewed, finalized and submitted the manuscript. All authors read and approved the final manuscript.

## Consent

Written informed consent was obtained from our patient for publication of this case report and any accompanying images. A copy of the written consent is available for review by the Editor-in-Chief of this journal.
